# An RNA editing fingerprint of cancer stem cell reprogramming

**DOI:** 10.1186/s12967-014-0370-3

**Published:** 2015-02-12

**Authors:** Leslie A Crews, Qingfei Jiang, Maria A Zipeto, Elisa Lazzari, Angela C Court, Shawn Ali, Christian L Barrett, Kelly A Frazer, Catriona HM Jamieson

**Affiliations:** Division of Regenerative Medicine, Department of Medicine, Moores Cancer Center at University of California, La Jolla, CA 92093 USA; Sanford Consortium for Regenerative Medicine, La Jolla, CA 92037 USA; Doctoral School of Molecular and Translational Medicine, Department of Health Sciences, University of Milan, Milan, Italy; Division of Genome Information Sciences, Department of Pediatrics, University of California, La Jolla, CA 92093 USA

**Keywords:** Cancer stem cells, Leukemia stem cells, RNA editing, Biomarkers, Leukemia, ADAR1

## Abstract

**Background:**

Deregulation of RNA editing by adenosine deaminases acting on dsRNA (ADARs) has been implicated in the progression of diverse human cancers including hematopoietic malignancies such as chronic myeloid leukemia (CML). Inflammation-associated activation of ADAR1 occurs in leukemia stem cells specifically in the advanced, often drug-resistant stage of CML known as blast crisis. However, detection of cancer stem cell-associated RNA editing by RNA sequencing in these rare cell populations can be technically challenging, costly and requires PCR validation. The objectives of this study were to validate RNA editing of a subset of cancer stem cell-associated transcripts, and to develop a quantitative RNA editing fingerprint assay for rapid detection of aberrant RNA editing in human malignancies.

**Methods:**

To facilitate quantification of cancer stem cell-associated RNA editing in exons and intronic or 3'UTR primate-specific Alu sequences using a sensitive, cost-effective method, we established an *in vitro* RNA editing model and developed a sensitive RNA editing fingerprint assay that employs a site-specific quantitative PCR (RESSq-PCR) strategy. This assay was validated in a stably-transduced human leukemia cell line, lentiviral-ADAR1 transduced primary hematopoietic stem and progenitor cells, and in primary human chronic myeloid leukemia stem cells.

**Results:**

In lentiviral ADAR1-expressing cells, increased RNA editing of MDM2, APOBEC3D, GLI1 and AZIN1 transcripts was detected by RESSq-PCR with improved sensitivity over sequencing chromatogram analysis. This method accurately detected cancer stem cell-associated RNA editing in primary chronic myeloid leukemia samples, establishing a cancer stem cell-specific RNA editing fingerprint of leukemic transformation that will support clinical development of novel diagnostic tools to predict and prevent cancer progression.

**Conclusions:**

RNA editing quantification enables rapid detection of malignant progenitors signifying cancer progression and therapeutic resistance, and will aid future RNA editing inhibitor development efforts.

**Electronic supplementary material:**

The online version of this article (doi:10.1186/s12967-014-0370-3) contains supplementary material, which is available to authorized users.

## Background

Dormant cancer stem cells (CSCs) are primary arbiters of cancer progression and a major focus of targeted therapy development efforts. Deregulation of RNA editing by adenosine deaminases acting on double-stranded (ds) RNA (ADARs) has been implicated in the progression of diverse human malignancies, including chronic and acute forms of leukemia [1-3], lobular breast cancer [4], hepatocellular carcinoma [5,6], and esophageal squamous cell carcinoma [7]. Recent evidence also supports a role for aberrant RNA editing in malignant reprogramming of progenitor cells into self-renewing CSCs, suggesting that ADARs and their target substrates may be harbingers of cancer progression [1,4,5].

Of particular relevance to the pathogenesis of human disease, over 90% of RNA editing occurs in primate-specific Alu sequences that form dsRNA secondary structures [8], often within non-coding regions such as introns and 3′UTRs [9]. Most RNA editing is carried out by ADAR-mediated C6 deamination of adenosines (A) to inosines (I) [10]. The ADAR family consists of three members, ADAR1 (*ADAR*), ADAR2 (*ADARB1*), and ADAR3 (*ADARB2*). In mouse hematopoietic development, ADAR1 plays a key role in hematopoietic stem cell (HSC) survival [11] and cell fate determination [12,13], and ADAR1 is the primary RNA editase expressed in human hematopoietic stem and progenitor cells [1]. At the transcript level, RNA editing can affect mRNA stability, localization, nuclear retention, and alternative splicing [14-16]. While RNA editing targets are relatively conserved in normal tissues [17], CSC-associated editing changes in response to malignant microenvironments could dramatically alter gene product stability and function. Additionally, aberrant RNA editing may drive stem cell regulatory transcript recoding and microRNA deregulation [18] leading to therapeutic resistance.

Advances in next-generation sequencing technologies and bioinformatics tools have led to the identification of hundreds of thousands of RNA editing sites throughout the human transcriptome [19,20], the majority of which are localized to hyper-edited regions [21]. With the availability of such massive new datasets, it is now critical to apply this knowledge to mine new and existing RNA-sequencing (RNA-seq) datasets of human tissues, to identify disease-relevant RNA editing loci. Previously, we found that human leukemia stem cells (LSC) from patients with blast crisis (BC) chronic myeloid leukemia (CML) harbored increased expression of ADAR1 compared with normal and chronic phase (CP) progenitors [1]. Since RNA editing may be selectively inhibited, it is of great clinical relevance to develop diagnostic and prognostic tools capable of accurately detecting fingerprints of aberrant RNA editing activity signifying cancer progression and therapeutic resistance. However, the functional role of RNA editing of individual transcripts, and its role in cancer progression and drug resistance, has not been widely addressed due to a lack of tools to quantify functionally relevant RNA editing events in a sensitive, cost-effective manner. Traditional Sanger sequencing is not sufficiently sensitive to detect editing events in rare stem cell regulatory transcripts, and transcriptome-wide profiling of RNA editing can be costly, technically challenging [22], and analysis requires expertise in specialized bioinformatics methods.

To address these challenges, we developed and applied a straightforward, clinically amenable assay to validate and quantify RNA recoding in stem cell regulatory transcripts identified through whole transcriptome RNA-seq analysis of purified primary LSC [1]. A site-specific RNA editing fingerprint of leukemic progression was validated in a lentiviral-ADAR1 model, and a sensitive RNA editing site-specific quantitative RT-PCR (RESSq-PCR) assay was devised to detect aberrant RNA editing in three different dsRNA contexts (3′UTRs, intronic Alu sequences, and coding exons). This clinically relevant assay sets the stage for RNA editing biomarker detection in diagnostic and prognostic assays for clinical use and as screening tools for identifying pharmacological modulators of RNA editing.

## Methods

### Primary samples and tissue processing

A large collection of leukemia patient samples and normal age-matched control bone marrow samples were obtained from consenting patients in accordance with Institutional Review Board approved protocols at UCSD and the University of Toronto (Additional file 1: Table S1). Peripheral blood or bone marrow samples were processed by Ficoll density centrifugation and viable cells stored in liquid nitrogen. Normal peripheral blood mononuclear cells (MNC) were obtained from AllCells (Alameda, CA). Mononuclear cells from control or CML patient samples were then further purified by magnetic bead separation of CD34^+^ cells (MACS; Miltenyi, Bergisch Gladbach, Germany) for subsequent FACS-purification of hematopoietic progenitor cells (CD34^+^CD38^+^Lin^-^) that represent the LSC fraction in BC CML [23]. Datasets from previous RNA-seq analyses of purified CML LSC are available through the NIH Sequence Read Archive (SRA), accession ID SRP028528.

### Primary CSC purification

For primary patient-derived LSC purification, CD34-selected cells were stained with fluorescent antibodies against human CD34, CD38, lineage markers (cocktail, all antibodies from BD Biosciences, San Diego, CA) and propidium iodide as previously described [1,23,24]. Following staining, cells were analyzed and sorted using a FACS Aria II (Sanford Consortium Stem Cell Core Facility), and hematopoietic progenitor (CD34^+^CD38^+^Lin^-^) populations were isolated. Freshly-sorted cells were collected in lysis buffer (Qiagen, Germantown, MD) for RNA extraction followed by RNA-seq or qRT-PCR analyses as previously described [1].

### High-fidelity PCR and Sanger sequencing analysis

For PCR and targeted Sanger sequencing analysis, 1-2 μL of first-strand cDNA templates were prepared for PCR in 25-50 μL reaction volumes using the high-fidelity KOD Hot Start DNA Polymerase kit according to the manufacturer’s instructions (EMD Millipore, Temecula, CA). “Outer” primers (Additional file 2: Table S2) used for sequencing produce predicted amplicons of approximately 150-250 nucleotides in length, and flank each editing site with approximately 50-100 bp on either side of the editing site to facilitate successful sequencing analysis. PCR cycling conditions were as follows: 95°C for 2 minutes, followed by 35 cycles of 95°C for 20 seconds, 62°C for 10 seconds and 70°C for 10 seconds, with a final extension step of 70°C for 30 seconds. Production of amplicons of the predicted size was verified for each outer primer set by DNA gel electrophoresis using 10-20 μL of the completed reaction mixture separated on 2% agarose gels containing ethidium bromide and visualized under UV light. Then, 15 μL of each reaction was processed within 24 hrs for PCR purification and sequencing was performed on ABI 3730 × l DNA Sequencers (Eton Bioscience, San Diego, CA). Sanger sequencing was carried out using the reverse outer primer used for PCR amplification, so edited loci are identified in the reverse complementary sequence as T/C nucleotides, except in cases where the gene products are transcribed from the reverse strand. Sequence chromatograms were analyzed using 4Peaks (by A. Griekspoor and Tom Groothuis, www.nucleobytes.com) and peak heights calculated using ImageJ (NIH). For RNA editing analysis of sequencing chromatograms, ratios of edited/WT peaks were calculated using the raw peak amplitude of each sequence trace.

### Cell lines and culture conditions

K562 cells (ATCC, Manassas, VA) were maintained in complete medium containing DMEM (Life Technologies, Carlsbad, CA), 10% fetal bovine serum (FBS), 1% Glutamax (Life Technologies), and 1% penicillin-streptomycin (Life Technologies). Parental cell lines and stably-transduced lines were authenticated as K562 by routine qRT-PCR analysis of BCR-ABL transcript levels [1]. Mouse bone marrow stromal cell lines (SL and M2) expressing human interleukin-3 (IL-3), stem cell factor (SCF) and granulocyte-colony stimulating factor (G-CSF), which support erythroid and myeloid cell expansion and differentiation, were maintained under standard culture conditions, as previously described [25]. Briefly, SL cells were grown in complete medium containing DMEM, 10% FBS, 1% Glutamax, and 1% penicillin-streptomycin, while M2 cells were grown in complete medium containing RPMI, 10% FBS, 1% Glutamax, and 1% penicillin-streptomycin (all from Life Technologies). Every four passages, cells were selected by addition of G418 and hygromycin to the culture media for one passage (3-4 days), to maintain human cytokine expression [25]. All cell lines were maintained in T-25 or T-75 culture flasks and were passaged at dilutions of 1:5-1:10 every 2-4 days. Low passage aliquots of cells were thawed every two months to ensure consistency of experiments.

### Lentiviral vector preparation and ADAR1 site-directed mutagenesis

We have previously characterized lentiviral vectors (Thermo Scientific) for overexpression of human ADAR1 p150-IRES-GFP [1]. For production of the catalytically-inactive ADAR1 mutated (ADAR1m) lentiviral vector, site-directed mutagenesis was carried out using the QuikChange II Site-Directed Mutagenesis Kit (Agilent) according to manufacturer’s instructions. Mutagenic primers were designed to produce a nucleotide substitution of A5293C, which generates an E912A amino acid change and abolishes RNA editase activity [26]. Primers contained the desired mutation and anneal to the same sequence on opposite strands of the plasmid (FW 5′-GTCAATGACTGCCATGCAGCAATAATCTCCCGG-3′, REV 5′-CCGGGAGATTATTGCTGCATGGCAGTCATTGAC-3′). XLI super competent cells were transformed with amplification products, after digestion with DpnI. Colonies were screened to identify mutated clones by DNA sequencing (Sanger sequencing, Eton Bioscience). Lentiviruses including control vectors (ORF) were produced according to established methods [27], with some batches of lentivirus being produced by the GT3 Viral Vector Core Facility (UCSD). We have previously validated lentivirus transduction efficiency in normal cord blood, 293 T cells and K562 cells, with an increase of approximately five-fold overexpression of ADAR1 transcripts confirmed by qRT-PCR analysis [1].

### Transduction of human cell lines and primary cells with lentiviral-ADAR1

For preparation of stably-transduced K562 cell lines, 50,000 wild-type (wt) K562 cells were plated into 96-well U-bottom plates in complete culture medium and transduced with lentiviral vectors expressing GFP (ORF), ADAR1-GFP, or ADAR1m-GFP at multiplicities of infection (MOI) from 50-200. After transduction, cultures were expanded for at least 5 passages and then processed for FACS purification of GFP-positive cells to establish pure stably-transduced lines. Stable expression of lentivirus-enforced ADAR1 conferring increased transcript levels of human ADAR1 in K562-ADAR1 cells was confirmed at every 5 passages by qRT-PCR.

For transduction of human normal HSC and CML progenitors, 50,000 CD34-selected (MACS, Miltenyi, Auburn, CA) cells were plated in 96-well U-bottom plates in StemPro media (Life Technologies) supplemented with human cytokines (IL-6 10 ng/mL, FLT3 ligand 50 ng/mL, SCF 50 ng/mL, and thrombopoietin 10 ng/mL) as previously described [1,24]. Twenty-four hours later, cells were transduced with lentiviral vectors (ADAR1 or ORF control, MOI = 50-100) for up to five days. For co-culture experiments, CD34-selected CP CML cells were transferred three days after transduction (MOI = 75) to monolayers of mouse bone marrow stromal cell cultures containing a 1:1 mixture of irradiated SL and M2 cells (50,000 total stromal cells per well in 24-well plates) [27]. Primary transduced cells were maintained in co-culture for five-days in Myelocult (Stem Cell Technologies, Vancouver, Canada) and then the total culture was harvested in lysis buffer for RNA extraction and qRT-PCR and RESSq-PCR analyses.

### Generation of a stable ADAR1 RNA editing detection model system

For purification of stably-transduced K562 cell lines, K562 cells transduced with lentiviral-ADAR1 or ORF controls (MOI = 50-200) were collected (minimum 1 × 10^6^ cells), washed in HBSS containing 2% FBS (staining media), and sorted using a FACS Aria II (Sanford Consortium Stem Cell Core Facility) for high GFP signal to purify the highly-transduced cell population. Purified cells were collected in complete media and maintained under routine culture conditions for K562 cells. The lentiviral-ORF and ADAR1 vectors include a blasticidin-resistance gene, but no significant change was observed in ADAR1 expression in stably-transduced cell lines following selection with blasticidin, and therefore no subsequent selection method was used after FACS purification. For all experiments, low-passage cells were thawed and maintained for no longer than two months in culture.

### Nucleic acid isolation, reverse transcription and quantitative RT-PCR

Cell lines, lentivirus-transduced primary hematopoietic cells, or FACS-purified primary cells were harvested in lysis buffer (Qiagen). RNA was purified using RNeasy extraction kits with a DNase (Qiagen) incubation step to digest any trace genomic DNA (gDNA) present. For RNA extraction from cell line lysates, 1-2 × 10^6^ cells were extracted using RNeasy mini columns, and for primary cells, 5-10 × 10^4^ cells were lysed and extracted using RNeasy micro columns. Genomic DNA was purified from equal numbers of cells lysed separately using the QIAamp DNA Blood Mini Kit (Qiagen) including an RNase A incubation step to digest any RNA present (Qiagen). RNA was stored at -80°C and gDNA stored at -20°C. Immediately prior to reverse transcription of RNA samples, nucleic acid concentrations were quantified on a NanoDrop 2000 spectrophotometer (Thermo Scientific), and purity was considered acceptable if A260/A280 values were ≥1.8. For standard qRT-PCR analysis of relative mRNA expression levels, DNA was synthesized using 50 ng - 1 μg of template RNA in 20 μL reaction volumes using the First-Strand SuperScript III Reverse Transcriptase Supermix (Life Technologies) followed by incubation with RNase H according to the manufacturer’s protocol and as described previously [24]. All cDNA products were stored at -20°C.

Because RNA editing events often occur in pre-processed RNA species, for cDNA preparation, three different conditions were evaluated, including (1) reverse transcription with gene-specific primers, (2) random hexamer primers only, or (3) a supermix containing both random hexamers and oligo-dT primers. Using cDNA prepared with all three methods was suitable for detection of intronic regions in cDNA prepared from DNase-digested RNA extracts, and allowed detection of increased RNA editing in K562-ADAR1 cells. We therefore proceeded with the standard supermix reverse transcription method for RESSq-PCR, as this would provide the most versatility for use of valuable human tissue samples and would allow analysis of total mRNA expression of other genes in the same samples.

We have made every effort to adhere to the Minimal Information for Publication of Quantitative Real-Time PCR Experiments (MIQE) guidelines [28]. Primers (Additional file 2: Table S2) were synthesized by ValueGene (San Diego, CA) and diluted to 10 μM working dilutions in DNase/RNase-free water. qRT-PCR was performed in duplicate using cDNA (1 μL reverse transcription product per reaction) on an iCycler (Bio-Rad, Hercules, CA) using SYBR GreenER Super Mix (Life Technologies) in 25-μL volume reactions containing 0.2 μM of each forward and reverse primer. Cycling conditions were as follows: 50°C for 2 minutes, then 95°C for 8 minutes and 30 seconds, followed by 40 cycles of 95°C for 15 seconds and 60°C for 60 seconds. Melting curve analysis was performed on each plate according to the manufacturer’s instructions. For standard qRT-PCR, HPRT mRNA transcript levels were used to normalize Ct values obtained for each gene, and relative expression levels were calculated using the 2^-ddCt^ method. To ensure validity of results, only Ct values <35 were used in gene expression analyses. All primer sets were tested in a no-template control (NTC) reaction containing only water instead of cDNA, and all gave Ct values >35 in NTC reactions. Production of a single amplicon of the expected size was verified for each primer set by DNA gel electrophoresis on 2% agarose gels containing ethidium bromide. For all cell line experiments, assays were repeated at least three times using separate RNA extracts and cDNA preparations.

### RNA editing fingerprint assay

In order to implement a rapid, cost-effective and clinically amenable method to detect a CSC-specific RNA editing fingerprint of cancer progression, we devised an RNA editing site-specific primer design strategy that is compatible with SYBR green qRT-PCR protocols (RESSq-PCR). Since RNA-edited transcripts are predicted to differ from wild-type (WT) sequences at only one nucleotide position, detection of RNA editing by qRT-PCR requires highly sensitive and selective primer design strategies. We have previously developed qRT-PCR primers that specifically recognize a gene product with a single point mutation (JAK2 V617F [29]), and here we employed a similar approach in designing RESSq-PCR primers. Allele-specific PCR strategies, based on positioning the 3′ base of a PCR primer to match one variant allele, have been used for the detection of SNPs and mutations in human gDNA or cDNA [30], however are not routinely used in quantitative detection of RNA single nucleotide modifications.

The RESSq-PCR assay primer design was applied to specific cancer and stem cell-associated loci (Table 1). Efficiency of all primer sets (Additional file 2: Table S2) was tested using serial dilutions of K562-ADAR1 cDNA. Primer sets were tested experimentally for human specificity and were considered to be human-specific if they returned Ct values >35 in cDNA prepared from mouse bone marrow stromal cell controls. Editing site-specific primers for some loci (Table 1) either failed to discriminate between cDNA and gDNA, or K562-ADAR1 cells did not display increased editing by Sanger sequencing, and therefore were not continued for assay development. RESSq-PCR was performed in duplicate using cDNA (1-5 μL reverse transcription product per reaction) or gDNA (10-200 ng input gDNA) on an iCycler (Bio-Rad) using SYBR GreenER Super Mix (Life Technologies) in 25-μL volume reactions containing 0.2 μM of each forward and reverse primer. Cycling conditions were the same as for standard qRT-PCR. Relative RNA editing rates (Relative edit/WT RNA) were calculated using the following calculation: 2^-(*Ct* Edit – *Ct* WT)^.

**Table 1:** **Chromosomal coordinates and regions of RNA editing biomarkers used for sequencing validation and RESSq-PCR assay development**

**Gene**	**Chr**	**Position**	**Region**	**Reference**
**MDM2**	12	69237534	3′UTR	[1,31]
**APOBEC3D**	22	39415872	Intron (Alu)	[1,31]
**GLI1**	12	57864624	Exon	[32]
**AZIN1**	8	103841636	Exon	[1,5]
SRP9	1	225976198	Intron (Alu)	[1,31]
SF3B3	16	70610885	3'UTR (Alu)	[1,31]
ABI1	10	27049636	Intron (Alu)	[1,31]
LYST	1	235990569	Intron (Alu)	[1,31]
MDM4	1	204521159	3'UTR (Alu)	[1,31]

Loci (hg19 chromosomal coordinates) in bold denote sites included in the RNA editing fingerprint assay for RESSq-PCR analysis.

### Statistical methods

qRT-PCR data were measured as a continuous outcome and each group was assessed for distribution. For normally distributed data, the Student’s *t*-test was applied to compare differences in RNA expression and editing ratios calculated by Sanger sequencing and RESSq-PCR, and values are expressed as means ± SEM. Experiments were performed in triplicate on blind-coded samples, and all statistical analyses were performed using GraphPad Prism (San Diego, CA).

## Results

### Selection and validation of aberrant RNA editing events associated with leukemia progression

**Figure 1:**
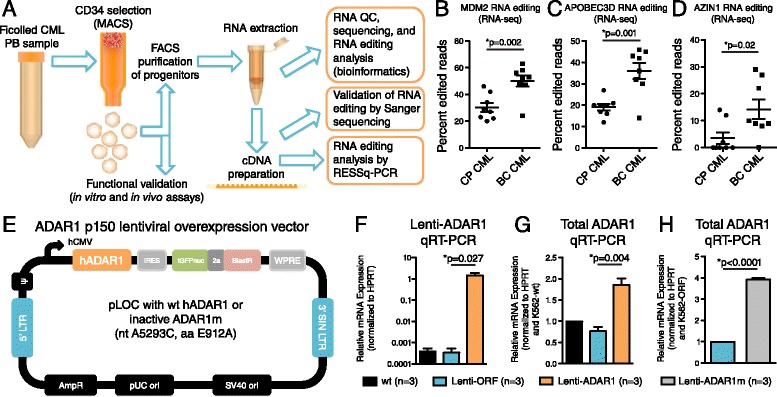
**Identification of an RNA editing fingerprint of malignant progenitor reprogramming, and stable ADAR1 overexpression in K562 cells. (A)** LSC purification strategy for detection of CSC-associated RNA recoding. **(B-D)** RNA-sequencing analysis of FACS-purified CP and BC CML LSC [1] showing A-to-G RNA editing changes in MDM2, AZIN1 and APOBEC3D (n = 8 per group). **(E)** Lentiviral construct expressing human ADAR1 or a catalytically inactive form (ADAR1m). **(F-H)** qRT-PCR analysis of cDNA prepared from K562 lines using primers detecting ADAR1 lentivirus **(F)** and total human ADAR1 **(G,H)** showing K562 leukemia cells stably transduced with active ADAR1 or inactive ADAR1m express high levels of ADAR1 transcripts compared with vector open reading frame (ORF) control backbone. *p < 0.05 by unpaired, two-tailed Student’s *t*-test.

Previous whole transcriptome analysis (Figure 1A) revealed widespread A-to-I RNA changes in CML LSC that were associated with malignant progenitor reprogramming typified by mis-splicing of key stem cell regulatory transcripts in selective niches during CML progression [1,27]. Whole transcriptome analysis of primary LSC identified 274 differentially edited sites in BC versus CP CML [1]. Candidate RNA editing loci were selected from this dataset to validate an RNA editing fingerprint of CML progression using a site-specific RNA editing detection assay. Eight LSC-specific loci (Table 1) were selected on the basis of greatest average fold-change in RNA editing frequency among significantly different sites (p < 0.005) [1]. These sites were located within transcripts of the ubiquitin ligase human homolog of mouse double minute 2 (MDM2); the cytidine deaminase apolipoprotein B mRNA editing enzyme, catalytic polypeptide-like 3D (APOBEC3D); antizyme inhibitor 1 (AZIN1); signal recognition particle 9 kDa (SRP9); splicing factor 3b, subunit 3 (SF3B3); abl-interactor 1 (ABI1); lysosomal trafficking regulator (LYST); and MDM4. Analysis of editing rates in individual samples from our RNA-seq dataset [1] showed increased RNA editing of MDM2, APOBEC3D, and AZIN1 in BC CML LSC compared with CP progenitors (Figure 1B-D). Notably, site-specific RNA editing of AZIN1 – a regulator of tumor growth [33] – causes exon recoding resulting in enhanced protein stability and is associated with aggressive hepatocellular carcinoma [5]. The majority of BC CML LSC samples displayed AZIN1 RNA editing, while RNA editing at this site was virtually undetectable in CP CML progenitor cells (Figure 1D). Consistent with these findings, Sanger sequencing of PCR products amplified with high-fidelity DNA polymerase confirmed increased APOBEC3D and AZIN1 RNA editing in BC versus CP CML progenitors (Additional file 3: Figure S1A-D).

### Development of an *in vitro* model of ADAR1-dependent RNA editing

To establish a cancer-relevant model system of augmented RNA editing, the BCR-ABL1-expressing human leukemia cell line K562 was stably transduced with lentiviral human ADAR1-GFP (K562-ADAR1); lentiviral ADAR1 mutant (A5293C) that lacks deaminase activity [26] (catalytically inactive, K562-ADAR1m); or vector open reading frame control (K562-ORF, Figure 1E). Transduced cells expressing high levels of GFP were FACS-purified to establish stable cell lines, and then expanded *in vitro*. ADAR1 expression was confirmed at regular intervals (approximately every 5 passages) by qRT-PCR (Figure 1F-H) using lentiviral-specific and human-specific primers (Additional file 2: Table S2).

### RNA editing site-specific qRT-PCR (RESSq-PCR) assay design and validation

**Figure 2:**
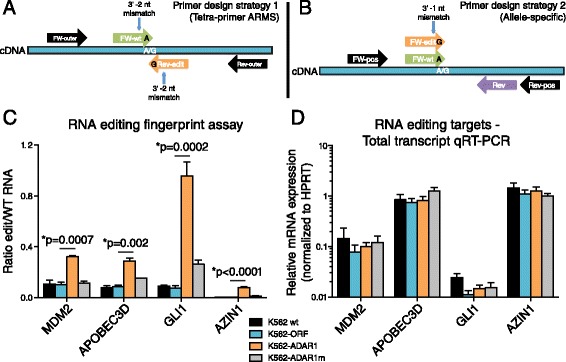
**RESSq-PCR assay primer design and RNA editing fingerprint validation in stable human ADAR1-overexpressing cells. (A,B)** Primer design strategy showing RNA editing site-specific qRT-PCR (RESSq-PCR) primer design strategy (1) to selectively detect wild-type **(A)** or edited (G/I) bases using the Tetra-primer amplification refractory mutation system (ARMS) principles **(A)**. Adaptation of the RESSq-PCR primer design strategy (2) for positions that are not compatible with the Tetra-primer ARMS method due to significant differences in GC content directly upstream and downstream or the edited nucleotide position **(B)**. FW = forward primer, Rev = reverse primer, Pos = positive control flanking primers. **(C)** RESSq-PCR analysis of MDM2, APOBEC3D, GLI1 and AZIN1 RNA recoding in stably-transduced K562-ADAR1 cells compared with K562 wt, K562-ORF and K562-ADAR1m lines (n = 2-4 per site). *p < 0.05 by unpaired, two-tailed Student’s *t*-test. **(D)** qRT-PCR analysis of MDM2, APOBEC3D, GLI1 and AZIN1 relative transcript expression using primers flanking each editing site in wild-type (wt) K562 (n = 5), K562-ORF (n = 5), K562-ADAR1 (n = 5) and K562-ADAR1m (n = 3) cDNA. For calculation of transcript control levels, Ct values were normalized to qRT-PCR Ct values using human-specific primers against HPRT.

A sensitive RNA editing fingerprint assay that employs a site-specific quantitative PCR (RESSq-PCR) strategy was devised to detect aberrant RNA recoding in three different dsRNA contexts (3′UTR, intronic Alu sequences, and coding exons). An additional known editing site within exon 10 of the stem cell self-renewal factor GLI1 was included because ADAR-directed RNA editing of this site promotes transcriptional activity of GLI1 protein. The edited form of GLI1 is less susceptible to inhibition by the negative regulator of hedgehog signaling, suppressor of fused (SUFU) [32], promoting stem cell-like behavior. For each RNA editing site, two sets of primers were designed: one pair detecting the WT transcript (an “A” base), and one pair detecting the edited transcript containing a “G” base representing inosine substitution (Figure 2A,B; Additional file 2: Table S2). Two primer design strategies were used to ensure highly specific detection of only A or G(I) alleles for each editing site. The primary design method applies the amplification refractory mutation system (ARMS) principle [34] and the tetra-primer ARMS-PCR web interface (Primer1 [30]) to design two unique primer sets per editing site that would generate unique PCR products for WT (A) or edited (G) transcripts. This strategy employs two outer and two inner primers for each editing site with melting temperatures ranging from 60-68°C. The forward (FW) outer and reverse (REV) outer primers flank the editing site and can be used for Sanger sequencing validation of each editing site, and also as a qRT-PCR positive control to ensure that the editing region is detectable in cDNA (Figure 2A). The 3′ ends of the FW inner and REV inner primers match either the WT A or edited G nucleotide, and an additional mismatch was incorporated two nucleotides upstream of the 3′ primer end to enhance allelic discrimination (Figure 2A), as previously described for quantitative detection of transcripts harboring single nucleotide genomic mutations [30].

A modified secondary primer design strategy was used in some cases where it was not possible to design primers with similar characteristics directly upstream and downstream of the editing site. This strategy employs a similar principle, however the FW inner primer sequences detecting WT and edited variants are almost identical, with only the 3′ nucleotide position differing to discriminate A or G bases (Figure 2B). An additional mismatch was incorporated one or two nucleotides upstream of the 3′ primer end to enhance allelic discrimination, and a common REV primer is used for both variants (Figure 2B). Due to the inherent restrictions of site-specific primer design, in some cases one set of primers was not human-specific or it was not possible to design FW inner and REV inner primers with similar characteristics in terms of melting temperature and GC content, and the second strategy was applied.

RESSq-PCR primers specific for edited sites in MDM2, APOBEC3D, GLI1 and AZIN1 distinguished G(I) bases in cDNA from K562-ADAR1 cells, with no signal in gDNA (Additional file 4: Figure S2A-D). In independent experiments, RESSq-PCR assays detected increased site-specific editing of MDM2, APOBEC3D, GLI1 and AZIN1 (Figure 2C). Relative A-to-G(I) editing ratios in MDM2, APOBEC3D, GLI1, and AZIN1 were increased by 2 to 10-fold in K562-ADAR1 cells, with only very low levels of editing detected when a mutated catalytically inactive ADAR1 construct was stably expressed at comparable levels (Figure 2C). Total transcript expression analysis of the RNA editing loci-containing regions measured by qRT-PCR using primers flanking the editing sites showed minimal differences in expression between groups (Figure 2D).

Sanger sequencing analysis of the stably-transduced K562-ADAR1 cell line confirmed increased A-to-G(I) changes in MDM2, APOBEC3D and GLI1 (Additional file 5: Figure S3), indicating that these sites are edited in an ADAR1-dependent manner. Notably, chromatograms from RNA editing-rich regions often show poor quality in heterogeneous cDNA preparations, as was observed for MDM2 (Additional file 5: Figure S3C). This precluded quantitative analysis of RNA editing allelic ratios by Sanger sequencing, whereas RESSq-PCR analysis reproducibly detected increased MDM2 editing in K562-ADAR1 cells (Figure 2C). Similar ADAR1-dependent increases in G versus A nucleotides in GLI1 were detected when comparing peak height ratios and RESSq-PCR, particularly when sequencing was performed using reverse primers (Additional file 5: Figure S3E, G; Figure 2C).

### Detection of lentiviral ADAR1-induced RNA editing in primary human cells

**Figure 3:**
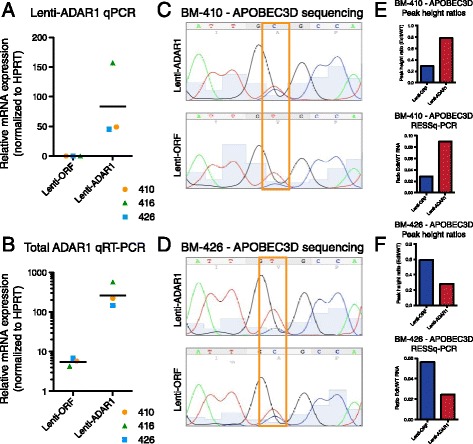
**Validation and quantification of RNA editing activity in primary bone marrow-derived hematopoietic stem and progenitor cells transduced with lentiviral-ADAR1.** CD34-selected cells from normal bone marrow (BM) samples (n = 3, average donor age = 64.3 ± 2.9 years old) were transduced with lentiviral (lenti)-ADAR1 or vector (ORF) control. After 4 days of culture, cells were lysed and processed for qRT-PCR and RESSq-PCR analysis. **(A,B)** Relative expression of lentivirus-derived (a) and total (b) ADAR1 levels in transduced BM samples (n = 3) showing increased human ADAR1 expression in ADAR1-transduced samples, with higher levels of total ADAR1 overexpression achieved in samples BM-410 and BM-416. **(C,D)** Representative Sanger sequencing analysis of high-fidelity PCR products amplified with primers flanking the APOBEC3D editing site showing increased G(I) peak in lenti-ADAR1 transduced cells that displayed robust ADAR1 expression (BM-410, C). **(E,F)** Quantification of sequencing peak height ratios and corresponding RESSq-PCR analysis in lenti-ORF and lenti-ADAR1 transduced BM samples.

To investigate whether lentiviral-ADAR1 expression alters RNA editing and can be detected by RESSq-PCR at CSC-associated sites in primary hematopoietic stem and progenitor cells, CD34^+^ cells were isolated from healthy human bone marrow (BM) or peripheral blood samples from CP CML patients. After 3-5 days *in vitro*, normal bone marrow cells transduced with lentiviral-ADAR1 displayed high levels of lentivirus-derived ADAR1 compared with ORF controls (Figure 3A,B). Sanger sequencing and RESSq-PCR analyses showed that while variable transduction efficiency appeared to greatly influence RNA editing rates, RNA editing activity measured by RESSq-PCR was routinely comparable to peak height ratios calculated by Sanger sequencing analysis (Figure 3C-F).

**Figure 4:**
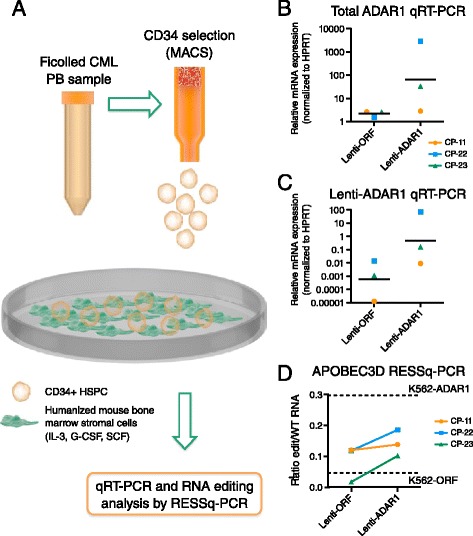
***In vitro*** **humanized stromal co-culture model and RESSq-PCR analysis of primary CP CML cells transduced with lentiviral-ADAR1. (A)** Schematic diagram of humanized bone marrow stromal co-culture assay. CD34-selected hematopoietic stem and progenitor cells (HSPC) isolated from patients with CP CML were transduced with lenti-ADAR1 or ORF control. After 3 days of culture, cells were transferred to SL/M2 mouse bone marrow stromal monolayers for co-culture and subsequent RESSq-PCR analysis. **(B,C)** Increased total ADAR1 **(B)** and lenti-ADAR1 **(C)** expression in transduced CP CML samples (n = 3). **(D)** RESSq-PCR analysis showing increased APOBEC3D RNA editing in lenti-ADAR1 transduced cells from patients with CP CML that harbored high ADAR1 expression after transduction. Horizontal dashed lines represent comparative RNA editing activity in K562-ADAR1 and K562-ORF cells.

In CP CML hematopoietic (CD34^+^) cells transduced with lentiviral-ADAR1 and co-cultured with humanized mouse stromal cells that recapitulate the bone marrow microenvironment [25] (Figure 4A), ADAR1 expression levels were variable across samples, with one sample displaying very high expression of ADAR1 after lentiviral-ADAR1 transduction (Figure 4B,C). Two out of three samples showed increased total ADAR1 expression after transduction compared with lentiviral-ORF controls, and this correlated with increased RNA editing of APOBEC3D detected by RESSq-PCR (Figure 4D).

### Detection of a CSC-specific RNA editing fingerprint of leukemic progression

**Figure 5:**
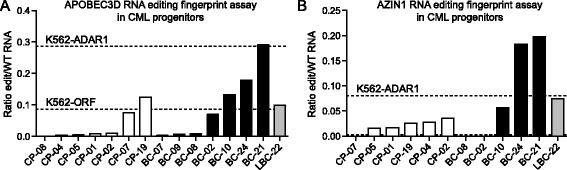
**Detection of increased RNA editing activity by RESSq-PCR analysis of primary chronic phase versus blast crisis CML progenitors.** RNA extracted from FACS-purified CD34^+^CD38^+^Lin^-^ primary CML progenitors was analyzed by RESSq-PCR to validate the RNA editing fingerprint of leukemic progression. **(A)** RESSq-PCR analysis detecting increased RNA editing in APOBEC3D in purified BC CML LSC versus CP progenitors. **(B)** RESSq-PCR analysis detecting increased RNA editing in AZIN1 in purified BC CML LSC versus CP progenitors. Horizontal dashed lines represent comparative RNA editing activity in K562-ADAR1 and K562-ORF cells.

To confirm the RNA editing fingerprint of leukemic progression by RESSq-PCR analysis in primary samples, FACS-purified progenitors from CP and BC CML patients (Additional file 1: Table S1) were analyzed. Remarkably, the majority of CP samples showed low levels of RNA editing at APOBEC3D and AZIN1 sites, while RNA editing in BC CML LSC was more variable and overall higher on average (Figure 5A,B), which was in agreement with RNA-seq and Sanger sequencing-based RNA editing analyses (Figure 1, Additional file 3: Figure S1). Together, these data demonstrate that quantitative RNA editing detection by RESSq-PCR provides a straightforward and robust measure of functional RNA editing activity, and shed new light on potential mechanisms of ADAR1-mediated generation of malignant progenitors that fuel therapeutic resistance, disease progression and relapse in CSC-driven malignancies.

## Discussion

The relative paucity of functional biomarkers that predict cancer progression represents a growing health care concern. This has provided the impetus for developing sensitive molecular detection systems to improve clinical diagnosis, prognostication, and patient stratification for clinical trials, by facilitating early detection of CSC that drive disease progression and therapeutic resistance. Alternative RNA splicing and editing of survival and self-renewal genes has been implicated in CSC generation and maintenance [1,24,27]. We previously reported that RNA editing is increased in LSC during CML progression, suggesting that RNA editing activity may be an important biomarker of CML progression [1,4], with relevance to a broad array of human malignancies. Specifically, ADAR1 was also highly expressed in pediatric acute lymphoblastic leukemias [2], hepatocellular carcinoma [5], esophageal squamous cell carcinoma [7], and in aggressive breast cancers [4]. Furthermore, a disrupted RNA editing balance is associated with poor prognosis for patients with hepatocellular carcinoma [6].

Next-generation technologies have enabled RNA and DNA sequencing of rare cell populations, providing insights into the gene networks and central pathways that contribute to cancer initiation, progression and therapeutic resistance. While a recent report described a multiplex PCR method to quantify RNA allelic ratios by sequencing at numerous sites [35], one obstacle to such analytical strategies is that high-throughput sequencing often must be performed by external rather than local laboratories. The RNA editing fingerprint assay described here can be performed using routine laboratory reagents and showed improved sensitivity over Sanger sequencing and traditional chromatogram analysis. Thus, RESSq-PCR, developed as an array-based technology detecting a CSC-specific RNA editing fingerprint of malignant reprogramming, could provide a rapid clinical assay for early detection of cancer progression and prognostication.

One of the most widely used traditional methods to detect RNA editing activity at specific sites is Sanger sequencing, however this technique can only detect RNA editing at sites where greater than 5-20% of transcripts are edited [36] and is best used to measure qualitative differences [37]. Because even low RNA editing rates may be functionally relevant to cancer [5], and differentially-edited loci in CML LSC show <5% editing in CP CML progenitors by RNA-seq [1], Sanger sequencing does not provide a sufficiently sensitive method for quantifying disease-associated RNA editing. A handful of strategies have been described to detect site-specific variant alleles by using WT (A) specific qRT-PCR primers [38] or custom Taqman probes [36], however the RESSq-PCR assay provides the first qRT-PCR strategy to detect edited human transcripts as biomarkers for CSC detection and disease stratification. Notably, RESSq-PCR can be used to detect endogenous RNA editing, while alternative strategies to detect RNA editing activity require transfection with exogenous substrates and reporter genes [39,40]. Although reporter tools are practical in screening assays to identify ADAR activators or inhibitors [40], minimal cellular manipulation is essential for future studies of the functional consequences of RNA editing modulation.

Notably, this straightforward nucleotide editing fingerprint assay could also be applied to detect cytidine deamination in RNA or DNA by APOBEC family proteins, which may contribute to DNA mutagenesis in human cancer [41-43]. Additionally, altered RNA editing was recently reported in neurodegenerative disease [44], and thus, methods for clinical detection of abnormal RNA editing could have wide-ranging applications for the diagnosis and treatment of a variety of degenerative, developmental and malignant disorders.

In the present study we noted that there may be some site-specific variability in RNA editing across patient samples and therefore in clinical applications it will be advantageous to evaluate multiple editing sites simultaneously using the RNA fingerprint assay. For example, sample BC-02 showed moderate editing at the APOBEC3D site by RESSq-PCR, but editing of AZIN1 was below the qRT-PCR detection threshold (Ct value >35) (Figure 5). Similarly, in our previous RNA-seq study (Figure 1B-D), at some sites a subset of BC samples showed low editing ratios, or editing activity within the range of CP samples, but showed higher editing rates at other sites. Thus, we predict that the prognostic significance of RNA editing activation may be in detection of one or more CSC-associated sites displaying increased editing rates.

Indeed, comparison of the RNA editing ratios in individual cases at each LSC-associated site determined previously by whole transcriptome sequencing revealed that while editing of AZIN1 was relatively low by RNA-seq in cases BC-02 and BC-08 (consistent with the RESSq-PCR results), these cases were among the ones that showed highest editing rates in MDM2 [1]. These site-specific differences are likely related to differential transcript stability after post-transcriptional processing [16], and highlight the importance of using a validated panel of RNA editing biomarkers to measure RNA editing activity clinically, rather than a single biomarker or ADAR1 expression levels alone.

Future studies evaluating larger cohorts of cancer patients, with comprehensive clinical annotation of patient treatment status and disease outcomes, will provide further insights into the relationship between aberrant RNA editing and risk for disease progression. Additionally, we hypothesize that RNA editing activity may be influenced by differing treatment status at the time of sample collection (e.g. hydroxyurea versus imatinib) and could also provide prognostic information related to risk for acquisition of drug resistance. Thus, assessment of sequential patient samples before and after treatment or disease progression will allow precise determination of the RNA editing levels that correlate with poorer prognosis in a variety of blood cancers and other CSC-driven malignancies.

## Conclusions

Recent evidence indicates that ADAR-directed RNA editing represents a novel mechanism of disease progression and a promising therapeutic target for diverse human cancers. Malignant RNA editing programs represent an unexplored resource for biomarker development, and the development of an RNA editing fingerprint assay demonstrates the application of novel findings from high-throughput sequencing and bioinformatics studies [1,27] to validate unique CSC-specific molecular signatures of cancer progression. Furthermore, the RESSq-PCR RNA editing diagnostic platform provides an innovative method for testing specificity of candidate CSC-targeted therapeutics to inhibit RNA editing, and could serve as an informative companion diagnostic for clinical trials. In summary, this study demonstrates the rapid translation of next-generation sequencing data into a functionally relevant tool for detection of an RNA editing fingerprint of malignant progenitor reprogramming that could identify patients at risk for cancer progression and therapeutic resistance.

## Additional files

Additional file 1: Table S1. Summary of normal bone marrow and CML chronic phase and blast crisis patient samples. Additional file 2: Table S2. Primers used for qPCR, RESSqPCR and direct sequencing analyses. Additional file 3: Figure S1. Sanger sequencing validation of intronic Alu-targeted RNA editing of APOBEC3D and exon-targeted RNA editing of AZIN1 in purified CML LSC. Additional file 4: Figure S2. Validation of RESSq-PCR primer specificity in K562-ADAR1 cDNA versus genomic DNA (gDNA). Additional file 5: Figure S3. Sanger sequencing analysis of APOBEC3D intronic Alu-targeted RNA editing, MDM2 3'UTR editing and exon-targeted RNA editing of GLI1 in K562-ADAR1 cells.

## Abbreviations

CSCs: Cancer stem cells
ADAR: Adenosine deaminases acting on double-stranded (ds) RNA
HSC: Hematopoietic stem cell
RNA-seq: RNA-sequencing
LSC: Leukemia stem cells
BC: Blast crisis
CML: Chronic myeloid leukemia
CP: Chronic phase
RESSq-PCR: RNA editing site-specific quantitative RT-PCR
MNC: Mononuclear cells
MOI: Multiplicity of infection
MDM2: Mouse double minute 2
APOBEC3D: Cytidine deaminase apolipoprotein B mRNA editing enzyme, catalytic polypeptide-like 3D
AZIN1: Antizyme inhibitor 1
SRP9: Signal recognition particle 9 kDa
SF3B3: Splicing factor 3b, subunit 3
ABI1: abl-interactor 1
LYST: Lysosomal trafficking regulator
ARMS: Amplification refractory mutation system
FW: Forward
REV: Reverse
