# Supplementary material for: An RNA editing fingerprint of cancer stem cell reprogramming
Source: J Transl Med. 2015 Feb 12;13:52. doi: 10.1186/s12967-014-0370-3 (PMC4341880; doi:10.1186/s12967-014-0370-3)
Supplement: Additional file 1: Table S1. — Summary of normal bone marrow and CML chronic phase and blast crisis patient samples. [file 12967_2014_370_MOESM1_ESM.pdf]

**Additional file 1: Table S1. Summary of normal bone marrow and CML chronic phase and blast crisis patient samples**

| Patient ID | Gender/ Age | Date | Disease Stage | WBC count ($K/mm^3$) | % Blast (PB) | Treatment | % Blast (BM) |
|---|---|---|---|---|---|---|---|
| NPB-1 | N/A | N/A | N/A | N/A | N/A | N/A | N/A |
| BM-410 | M/66 | 13-Jun-12 | NBM | N/A | N/A | None | N/A |
| BM-416 | F/66 | 26-Jun-12 | NBM | N/A | N/A | None | N/A |
| BM-426 | M/61 | 3-Jul-12 | NBM | N/A | N/A | None | N/A |
| CP-01 | M/60 | 13-Nov-08 | CP | 189 | <5 | None | <5 |
| CP-02 | F/63 | 23-May-08 | CP | 326 | 5 | None | <5 |
| CP-04 | M/44 | 14-Oct-08 | CP | 306 | 5.8 | None | N/A |
| CP-05 | M/26 | 21-Sep-09 | CP | 231 | <1 | None | <5 |
| CP-07 | F/25 | 25-Mar-03 | CP | 134 | 0 | N/A | N/A |
| CP-08 | M/40 | 27-Jan-95 | CP | 381 | 7.6 | None | N/A |
| CP-11 | M/39 | 30-Oct-08 | CP | 689 | <5 | None | <5 |
| CP-12 | N/A | 26-Aug-09 | CP | 390 | <5 | None | N/A |
| CP-19 | M/40 | 20-Oct-10 | CP | 221 | 13 | None | <5 |
| CP-22 | M/54 | 8-May-12 | CP | 95 | <1 | None | <5 |
| CP-23 | M/53 | 17-Apr-13 | CP | 68 | N/A | None | N/A |
| BC-02 | M/34 | 26-Aug-04 | BC | 241 | 92 | None | 90 |
| BC-07 | M/48 | 29-Oct-93 | BC | 209 | 86 | Hydroxyurea | N/A |
| BC-08 | M/53 | 27-Jul-00 | BC | 98 | 83 | Hydroxyurea | 90 |
| BC-09 | M/65 | 17-Oct-91 | BC | 72 | 42 | None | 90 |
| BC-10 | M/40 | 21-Sep-93 | BC | 133 | 82 | None | N/A |
| BC-19 | M/46 | 23-Nov-07 | BC | 127 | 30 | Imatinib followed by dasatinib | 40-50 |
| BC-21 | M/41 | 10-Apr-09 | AP | N/A | N/A | N/A | N/A |
| LBC-22 | N/A | 19-May-08 | LBC | N/A | N/A | N/A | N/A |
| BC-24 | N/A | 23-Feb-06 | BC | N/A | N/A | N/A | N/A |
| BC-25 | M/58 | 21-Jun-09 | BC | 9 | 32 | Imatinib & Hydroxyurea | N/A |

Samples were collected prior to treatment except for samples noted in the table that received treatment with hydroxyurea and/or tyrosine kinase inhibitor therapy with imatinib/dasatinib. NPB = normal peripheral blood, NBM = normal bone marrow, CP = chronic phase CML, AP = accelerated phase CML, BC = blast crisis CML, LBC = lymphoid blast crisis, N/A = data not available.
