# Supplementary material for: An RNA editing fingerprint of cancer stem cell reprogramming
Source: J Transl Med. 2015 Feb 12;13:52. doi: 10.1186/s12967-014-0370-3 (PMC4341880; doi:10.1186/s12967-014-0370-3)
Supplement: Additional file 2: Table S2. — Primers used for qPCR, RESSqPCR and direct sequencing analyses. [file 12967_2014_370_MOESM2_ESM.pdf]

Additional file 2: Table S2. Primers used for qPCR, RESSqPCR and direct sequencing analyses

**Standard qPCR**	**Primer Set**	**FW 5'-3'**	**REV 5'-3'**	**Amplicon length**	**Primer location**
Lenti-ADAR1	Total	AAAAAGCAGGCTCCACCAT	ACGGTGTCTGCTTTCCAATC	205 bp	Vector
Human ADAR1	Total	TGCTGCTGAATTCAAGTTGG	TCGTTCTCCCCAATCAAGAC	171 bp	Exon
ADAR1 p150	Isoform	AACGAAAGCGAAATTGAACC	GGGTGTAGTATCCGCTGAGG	238 bp	Exon
ADAR1 p110	Isoform	GACTGAAGGTAGAGAAGGCTACG	TGCACTTCCTCGGGACAC	100 bp	Exon
ADAR2	Total	TGTTCCGTGTGTGTCCAGTT	CGGCAGGTCAGAGTTTTCTC	179 bp	Exon
Human HPRT	Total	TCAGGGATTTGAATCATGTTTGTG	CGATGTCAATAGGACTCCAGATG	111 bp	Exon
**RESSq-PCR**	**Primer Set**	**FW 5'-3'**	**REV 5'-3'**		
MDM2	Outer (Seq)	ATAGGACTGAGGTAATTCTGCACAGCA	AAGAGATTCTGCTTGGTTGTAGCTGAAG	182 bp	3’UTR
	WT	ATAGGACTGAGGTAATTCTGCACAGCA	ATAATGCTTGGAGGACCTCCACATGT	139 bp	3’UTR
	Edit	TAAATGGCCAAAGGGATTAGTAGTGTG	AAGAGATTCTGCTTGGTTGTAGCTGAAG	96 bp	3’UTR
APOBEC3D	Outer (Seq)	CTCTGGGATCTCTCTGCCTCCAAATATC	GAGGCTGAAGCAGAAGAATCGCTTAAAC	161 bp	Intron
	WT	GTCCAGGCTGGAATGCAATGTCA	GAGGCTGAAGCAGAAGAATCGCTTAAAC	85 bp	Intron
	Edit	CTCTGGGATCTCTCTGCCTCCAAATATC	GAGGTTGCAGTGAGTCCAGATGGC	123 bp	Intron
Gli1	Outer (Seq)	GGGGAGGACAGAACTTTGATCCTTACCT	AAGTCCATATAGGGGTTCAGACCACTGC	160 bp	Exon
	WT	GGGGAGGACAGAACTTTGATCCTTACCT	CTGGCTCTTCCTGTAGCCCGCT	111 bp	Exon
	Edit	ACTGAGAATGCTGCCATGGATGATG	AAGTCCATATAGGGGTTCAGACCACTGC	96 bp	Exon
AZIN1	Outer (Seq)	ACTGAATGACATCATGTAATAAATGGCT	AATACAAGGAAGATGAGCCTCTGTTTAC	209 bp	Exon
	WT	CATTCAGCTCAGGAAGAAGACATCT	AATACAAGGAAGATGAGCCTCTGTTTAC	103 bp	Exon
	Edit	ACTGAATGACATCATGTAATAAATGGCT	GAGCTTGATCAAATTGTGGCAG	153 bp	Exon
SRP9	Outer (Seq)	CACTGTCTCAAAAATACATACCTTCAGCA	CTAAAAATACAAAAATTAGCTGGGCGTG	220 bp	Intron
SF3B3	Outer (Seq)	TCACACCTGTAATCCCAGCACTTTGA	ATAGGCTGAAGTGATCCTCCTGTCTCAG	191 bp	3’UTR
ABI1	Outer (Seq)	AGAGAGCCAAGAAATAAGCCTTTAAGGG	GTTCAAGTGATTCTCCTGTCTCAACCTC	231 bp	Intron
LYST	Outer (Seq)	TGGCAAAACCCTGTTTCTACTAAAAATAT	TTTGAGACAGAGTCTCACTTTTTCACC	191 bp	Intron
MDM4	Outer (Seq)	TGGAAGAAGAATTGTCTTGGACCACACA	GTAAAAATGGGGTTTCTCCATGTCGGTC	192 bp	3’UTR

For outer primers used for qPCR and Sanger sequencing (Seq), primers were designed to flank editing sites (approximately 50-100 nucleotides upstream and downstream of the editing site).
