# Supplementary material for: An RNA editing fingerprint of cancer stem cell reprogramming
Source: J Transl Med. 2015 Feb 12;13:52. doi: 10.1186/s12967-014-0370-3 (PMC4341880; doi:10.1186/s12967-014-0370-3)
Supplement: Additional file 3: Figure S1. — Sanger sequencing validation of intronic Alu-targeted RNA editing of APOBEC3D and exon-targeted RNA editing of AZIN1 in purified CML LSC. [file 12967_2014_370_MOESM3_ESM.pdf]

Additional file 3: Figure S1

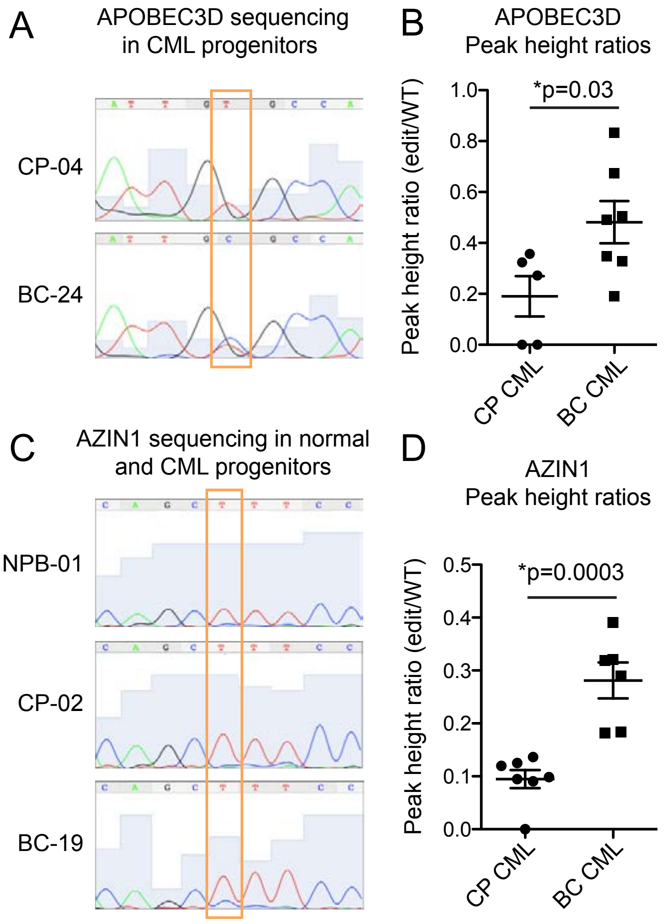

Figure S1. Sanger sequencing validation of intronic Alu-targeted RNA editing of APOBEC3D and exon-targeted RNA editing of AZIN1 in purified CML LSC
cDNA from FACS-purified $CD34^{+}CD38^{+}Lin^{-}$ normal peripheral blood-derived (NPB) hematopoietic progenitor cells and CP and BC CML LSC was amplified by high-fidelity PCR using primers flanking LSC-associated RNA editing sites in APOBEC3D and AZIN1, following by Sanger sequencing analysis. (A, B) Representative chromatograms and edit (C/G=I) versus wild-type (T/A) peak height ratio quantification (ImageJ) from Sanger sequencing analysis of high-fidelity PCR products amplified with primers flanking the APOBEC3D editing site in CP (n=5) and BC (n=8) CML LSC cDNA. (C, D) Representative chromatograms and edit (C/G=I) versus wild-type (T/A) peak height ratio quantification (ImageJ) from Sanger sequencing analysis of high-fidelity PCR products amplified with primers flanking the AZIN1 editing site in NPB (n=1), CP (n=7) and BC (n=6) CML LSC cDNA. *$p<0.05$ by unpaired, two-tailed Student's t-test.
