# Supplementary material for: An RNA editing fingerprint of cancer stem cell reprogramming
Source: J Transl Med. 2015 Feb 12;13:52. doi: 10.1186/s12967-014-0370-3 (PMC4341880; doi:10.1186/s12967-014-0370-3)
Supplement: Additional file 4: Figure S2. — Validation of RESSq-PCR primer specificity in K562-ADAR1 cDNA versus genomic DNA (gDNA). [file 12967_2014_370_MOESM4_ESM.pdf]

Additional file 4: Figure S2

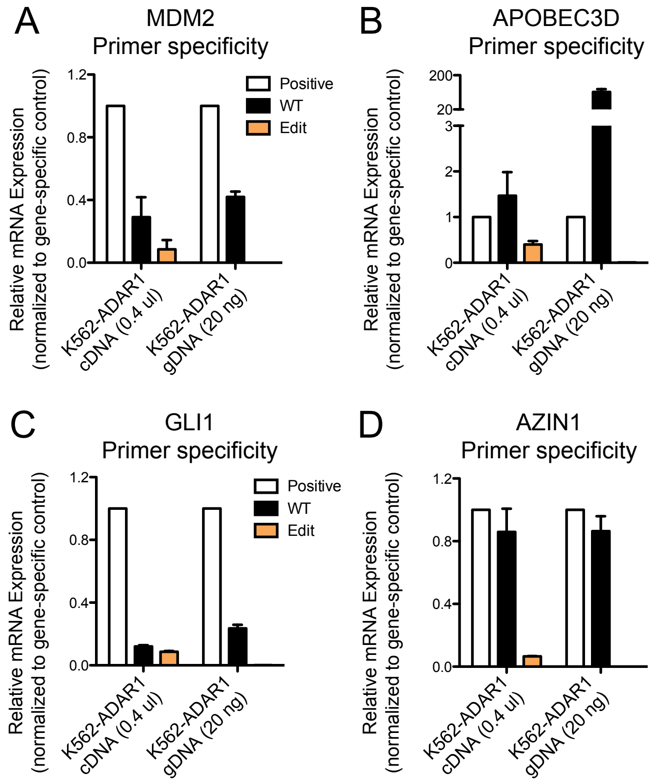

Figure S2. Validation of RESSq-PCR primer specificity in K562-ADAR1 cDNA versus genomic DNA (gDNA)

RESSq-PCR analysis was carried out to evaluate specificity of primers detecting edited MDM2, APOBEC3D, GLI1 and AZIN1 transcripts. (A-D) Relative expression of RNA-edited (G/I) and wild-type (WT, A) transcripts of MDM2 (A), APOBEC3D (B), GLI1 (C) and AZIN1 (D) in cDNA and gDNA from K562-ADAR1 cells. All Ct values were normalized to transcript-specific controls amplified using primers flanking each editing site. Ct values for edit-specific primers were all >35 in gDNA.
