# Supplementary material for: An RNA editing fingerprint of cancer stem cell reprogramming
Source: J Transl Med. 2015 Feb 12;13:52. doi: 10.1186/s12967-014-0370-3 (PMC4341880; doi:10.1186/s12967-014-0370-3)
Supplement: Additional file 5: Figure S3. — Sanger sequencing analysis of APOBEC3D intronic Alu-targeted RNA editing, MDM2 3'UTR editing and exon-targeted RNA editing of GLI1 in K562-ADAR1 cells. [file 12967_2014_370_MOESM5_ESM.pdf]

Additional file 5: Figure S3

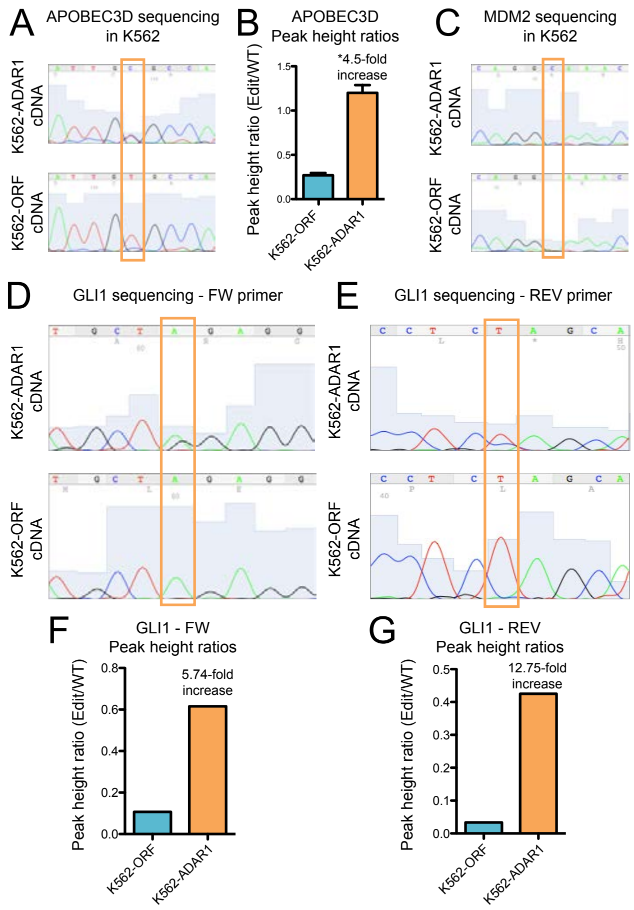

Figure S3. Sanger sequencing analysis of APOBEC3D intronic Alu-targeted RNA editing, MDM2 3'UTR editing and exon-targeted RNA editing of GLI1 in K562-ADAR1 cells
cDNA samples from stably-transduced K562 cell lines expressing ADAR1 or vector (ORF) control were subjected to high-fidelity PCR using primers flanking RNA editing sites in APOBEC3D, MDM2 or GLI1 and analyzed by Sanger sequencing or RESSq-PCR. (A) Representative chromatograms from Sanger sequencing analysis of PCR products amplified with primers flanking the APOBEC3D editing site and sequenced using a reverse primer. A robust C (G=I) peak was observed in K562-ADAR1 cells that was virtually undetectable in K562-ORF control cells. (B) Quantification of peak height ratios in K562-ORF and K562-ADAR1 cells. Peak height ratios of edit (C/G) versus wild-type (WT, T/A) nucleotides calculated using ImageJ (n=2). (C) Representative Sanger sequencing analysis of high-fidelity PCR products amplified with primers flanking the MDM2 editing site. (D, E) Representative Sanger sequencing analysis of high-fidelity PCR products amplified with primers flanking the GLI1 editing site showing increased G/C (=I) peaks in K562-ADAR1 cells that were not detectable in K562-ORF control cells. Representative sequencing results are shown using both forward (FW, D) and reverse (REV, E) primers. (F, G) Quantification of peak height ratios reflecting GLI1 editing rates in K562-ORF and K562-ADAR1 cells from chromatograms shown in panels D and E. *$p<0.05$ by unpaired, two-tailed Student's t-test compared to K562-ORF controls.
